# Enhancing diagnosis and treatment of superior cluneal nerve entrapment: cadaveric, clinical, and ultrasonographic insights

**DOI:** 10.1186/s13244-023-01463-0

**Published:** 2023-07-03

**Authors:** Wei-Ting Wu, Kamal Mezian, Ondřej Naňka, Lan-Rong Chen, Vincenzo Ricci, Chih-Peng Lin, Ke-Vin Chang, Levent Özçakar

**Affiliations:** 1 Department of Physical Medicine and Rehabilitation, National Taiwan University Hospital, Bei-Hu Branch, No. 87, Nei-Jiang Rd., Wan-Hwa District, Taipei, Taiwan; 2 Department of Physical Medicine and Rehabilitation, National Taiwan University College of Medicine, Taipei, Taiwan; 3 Department of Rehabilitation Medicine, Charles University, First Faculty of Medicine and General University Hospital in Prague, Prague, Czech Republic; 4 Institute of Anatomy, Charles University, First Faculty of Medicine, Prague, Czech Republic; 5 Physical and Rehabilitation Medicine Unit, Luigi Sacco University Hospital, ASST Fatebenefratelli-Sacco, Milan, Italy; 6 Department of Anesthesiology, National Taiwan University Hospital and National Taiwan University College of Medicine, Taipei, Taiwan; 7 Center for Regional Anesthesia and Pain Medicine, Wang-Fang Hospital, Taipei Medical University, Taipei, Taiwan; 8 Department of Physical and Rehabilitation Medicine, Hacettepe University Medical School, Ankara, Turkey

**Keywords:** Low back pain, Neuropathy, Sonography, Dextrose, Hydro-dissection

## Abstract

**Objectives:**

Low back pain is a prevalent public health issue caused by superior cluneal nerve (SCN) entrapment. This study aimed to explore the course of SCN branches, cross-sectional area (CSA) of the nerves, and effects of ultrasound-guided SCN hydrodissection.

**Methods:**

SCN distance relative to the posterior superior iliac spines was measured and compared with ultrasound findings in asymptomatic volunteers. The CSA of the SCN, pressure-pain threshold, and pain measurements were obtained from asymptomatic controls and patients with SCN entrapment at various time points after hydrodissection (with 1 mL of 50% dextrose, 4 mL of 1% lidocaine, and 5 mL of 1% normal saline) in the short-axis view.

**Results:**

Twenty sides of 10 formalin-fixed cadavers were dissected. The SCN locations on the iliac crest did not differ from the ultrasound findings in 30 asymptomatic volunteers. The average CSA of the SCN across different branches and sites ranged between 4.69–5.67 mm^2^ and did not vary across different segments/branches or pain statuses. Initial treatment success was observed in 77.7% (*n* = 28) of 36 patients receiving hydrodissection due to SCN entrapment. A group with initial treatment success experienced symptom recurrence in 25% (*n* = 7) of cases, and those with recurrent pain had a higher prevalence of scoliosis than those without symptom recurrence.

**Conclusions:**

Ultrasonography effectively localizes SCN branches on the iliac crest, whereby increased nerve CSA is not useful for diagnosis. Most patients benefit from ultrasound-guided dextrose hydrodissection; however, those with scoliosis may experience symptom recurrence and whether structured rehabilitation can reduce recurrence post-injection should be considered as one perspective in future research.

*Trial registration* ClinicalTrials.gov (NCT04478344). Registered on 20 July 2020, https://clinicaltrials.gov/ct2/show/NCT04478344?cond=Superior+Cluneal+Nerve&cntry=TW&draw=2&rank=1.

**Critical relevance statement** Ultrasound imaging accurately locates SCN branches on the iliac crest, while enlargement of the CSA is not useful in diagnosing SCN entrapment; however, approximately 80% of SCN entrapment cases respond positively to ultrasound-guided dextrose hydrodissection.

**Graphical abstract:**

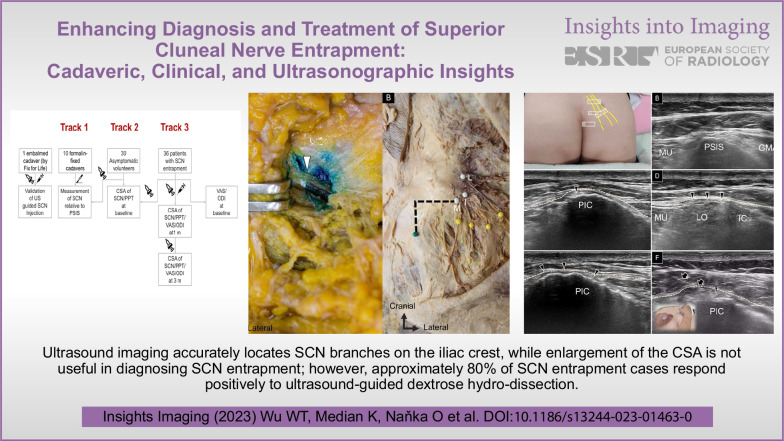

**Supplementary Information:**

The online version contains supplementary material available at 10.1186/s13244-023-01463-0.

## Key Points

- Ultrasound imaging accurately locates SCN branches on the iliac crest.
- Enlargement of the CSA is not useful in diagnosing SCN entrapment.
- Approximately 80% SCN entrapment cases respond positively to ultrasound-guided dextrose hydrodissection.
- Scoliosis is associated with symptom recurrence in patients after successful injection.

**Supplementary Information:**

The online version contains supplementary material available at 10.1186/s13244-023-01463-0.

## Introduction

Low back pain has become a significant public health issue in recent years, with an age-standardized prevalence between 7.50–8.20% [1]. Despite being commonly overlooked or misdiagnosed during clinical practice, superior cluneal nerve (SCN) entrapment could account for up to 14% of cases with low back pain [2]. The SCN is the lateral cutaneous division of the L1–3 spinal nerve dorsal rami; it typically has three branches that innervate the skin of the upper buttock [3]. After piercing the erector spinae muscle to course underneath the thoracolumbar fascia, the medial branch of the SCN commonly passes through an osteofibrous tunnel on the iliac crest, where it is most commonly entrapped [4]. The usual presentation of SCN entrapment includes tenderness over the middle portion of the posterior iliac crest with pain radiating to the proximal gluteal region. The standard treatment for SCN entrapment involves nerve block with local anesthetics, whereas surgical release of the thoracolumbar fascia may be necessary in recalcitrant cases [5].

High-resolution ultrasound allows for the visualization of the SCN and relevant pathologies, such as neuritis, after posterior lumbar interbody fusion surgery [6, 7]. As a cutaneous nerve, a short-axis view of the terminal portion of the SCN is easily visible as a circular hypoechoic fascicle within a hyperechoic adipose background [8]. Notably, the SCN can be traced from the subcutaneous layer to the superficial fascia overlying the erector spinae using a distal-to-proximal approach. Although measurement of its cross-sectional area (CSA) is feasible, no studies have examined the usefulness of ultrasound-derived CSA for diagnosing SCN entrapment or for ultrasound-guided localization of the SCN on the iliac crest [5].

Ultrasound-guided SCN injections have been described to provide postsurgical analgesia for the upper buttock [9] or relieve painful SCN entrapment. To achieve the former, the fascial plane between the thoracolumbar fascia and erector spinae can be further fully hydro-dissected with local anesthetic infiltration [10]. For the latter, the target should be chosen close to the nerve's most compressive point, usually on the iliac crest [11]. A clinical trial is still needed to evaluate the effectiveness of ultrasound-guided injection for SCN entrapment. Accordingly, this study used cadaveric models, asymptomatic volunteers, and patients with SCN entrapment to explore (i) the course of SCN branches over the iliac crest, (ii) the SCN CSA at different levels and its diagnostic value, and (iii) the effects of ultrasound-guided hydrodissection and the prognosis thereafter.

## Materials and methods

### Ethical approval

**Fig. 1:**
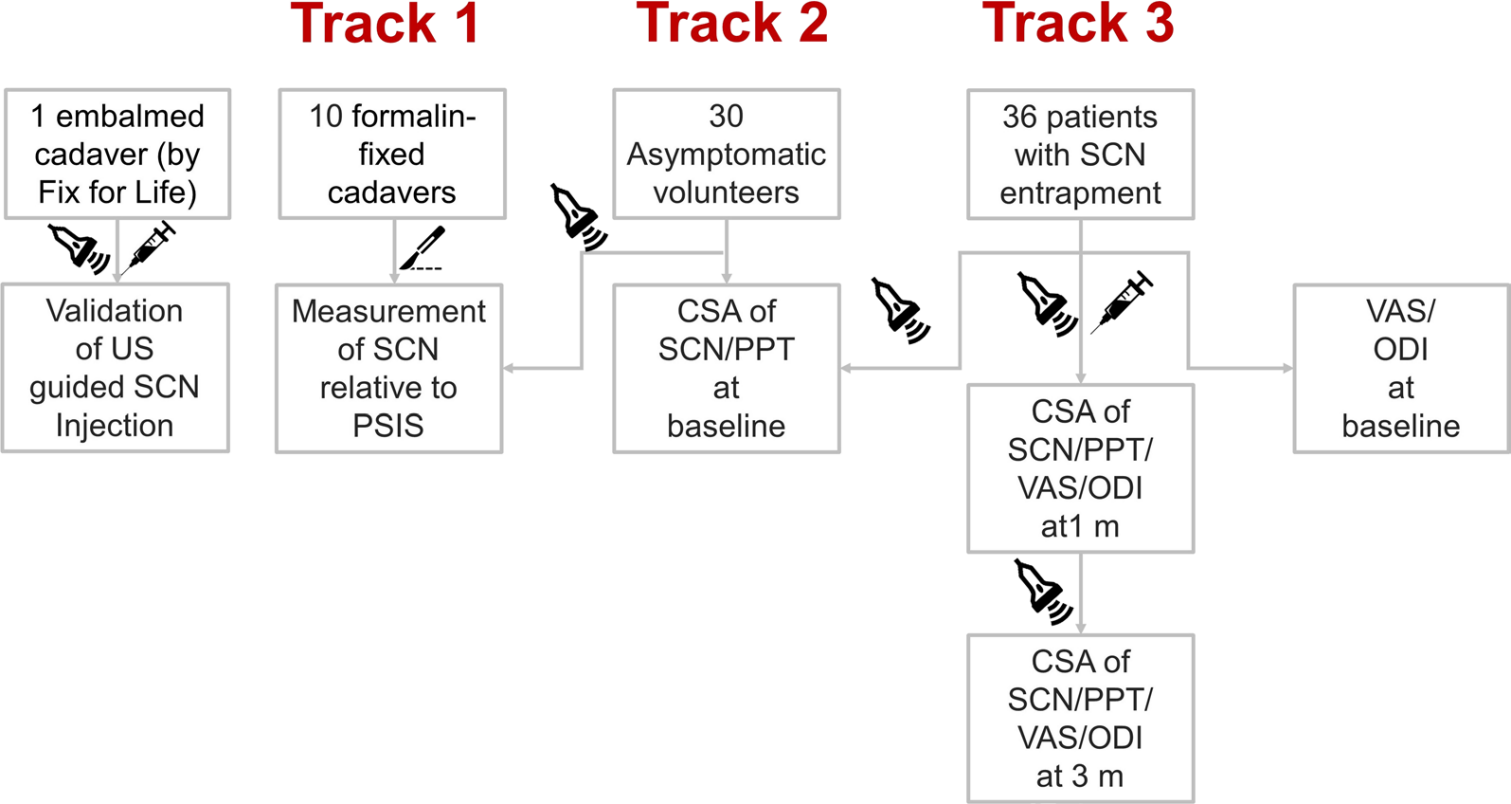
Flow diagram of the cadaveric study (track 1), human volunteer study (track 2) and clinical trial (track 3) of ultrasound (US) guided injection for the superior cluneal nerves (SCN). PSIS, posterior superior iliac spine; CSA, cross-sectional area; PTT, pain pressure threshold; VAS, visual analogue scale of pain; ODI, Oswestry Disability Index

Cadaver models were investigated with the approval of the Institute of Anatomy, First Faculty of Medicine, Charles University, Prague. Informed consent was obtained from healthy volunteers and patients. The study was approved by the Institutional Review Board of the National Taiwan University Hospital (201912037RINC) and registered at ClinicalTrials.gov (NCT04478344). The study flow is illustrated in Fig. 1.

### Techniques of ultrasound imaging and guided injection

**Fig. 2:**
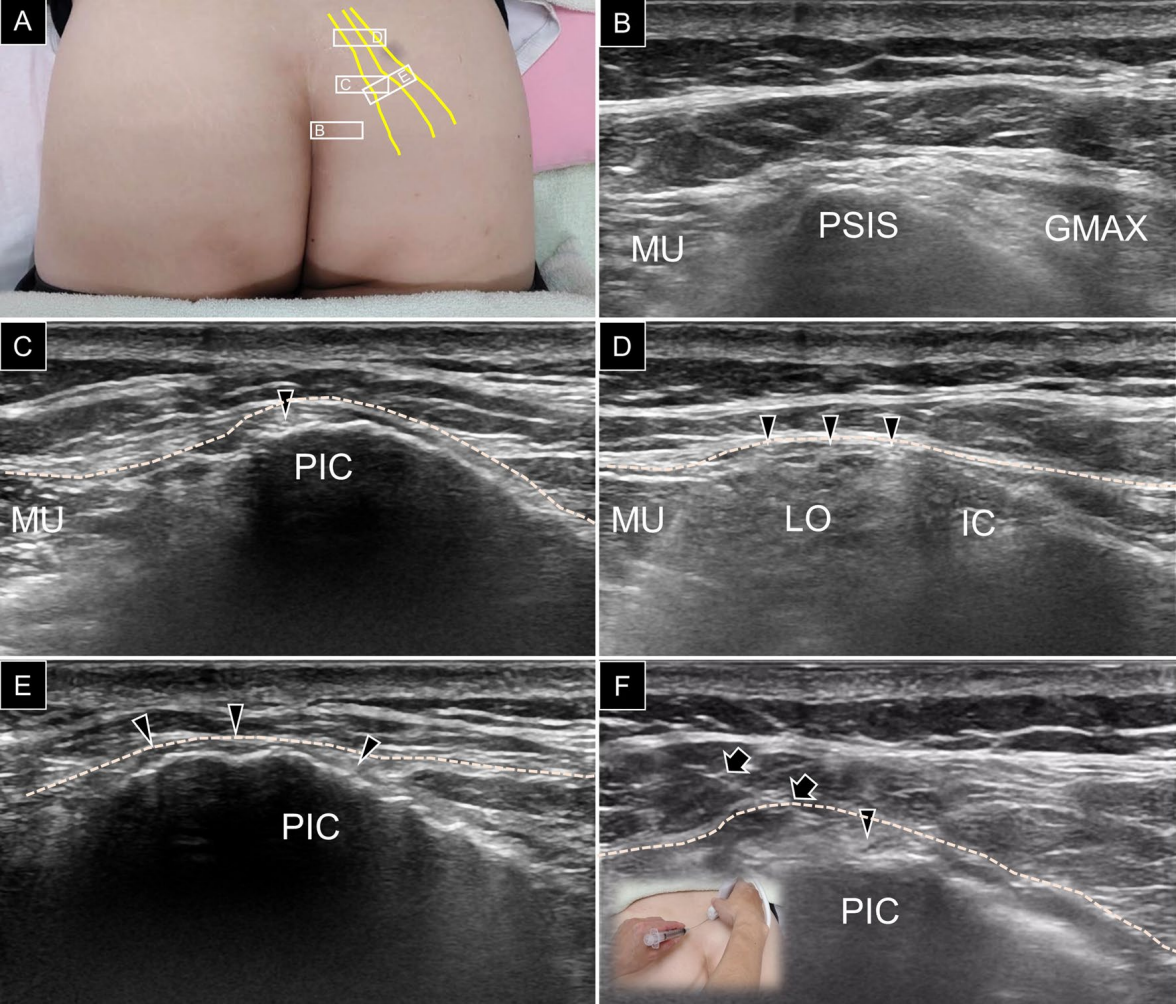
**A** A, B, C and D inside the squares correspond to probe positioning for the subgraphs of A, B, C and D for scanning of the superior cluneal nerves (yellow lines and black arrowheads). **B** The probe is placed in the horizontal plane over the posterior superior iliac spine (PSIS) and (**C**) is then relocated more cranially to see the medial branch of the superior cluneal nerve embedded underneath the thoracolumbar fascia (white dashed line). **D** Moving the probe more cranially, the superior cluneal nerve is seen over the erector spinae muscle. **E** Shifting the probe more laterally, the three branches of the superior clunear nerve are identified along the posterior iliac crest (PIC). **F** Ultrasound guided hydrodissection is performed for the superior cluneal nerves using the in-plane, medial-to-lateral approach. MU, multifidus; GMAX, gluteus maximus; LO, longissimus; IC, iliocostalis; arrows, needle trajectory

A board-certified physiatrist who had been practicing musculoskeletal ultrasound for > 10 years conducted the scanning and guided injections using a linear probe at 5–18 MHz (HI VISION, Ascendus, Hitachi). Participants were placed in the prone position with their lumbar and gluteal regions exposed (Fig. 2A). First, the posterior superior iliac spine (PSIS) was located in the axial plane (Fig. 2B), and the ultrasound transducer was gradually moved in the cranial direction. As the gluteus maximus muscle faded away and the gluteus medius muscle emerged, a concavity on the iliac crest was visualized, representing the fibro-osseous tunnel that harbors the medial branch of the SCN (Fig. 2C) [11]. The transducer was then relocated more proximally to visualize the nerve situated between the thoracolumbar fascia and erector spinae (typically the multifidus and longissimus) muscles (Fig. 2D) [10].

Once the course of the medial SCN branch was determined, the transducer was shifted laterally and repositioned at the PSIS level. The medial end of the transducer was pivoted toward the S3 or S4 foramen to obtain a better axial view of the intermediate and lateral branches located in the subcutaneous tissue over the gluteus medius muscle. By moving the transducer cranially, the two aforementioned branches could be seen traveling across the iliac crest (Fig. 2E) [8]. The locations of the nerves were validated by provoking tingling sensations following the application of percutaneous electric stimulation using a Stimuplex HNS 12 nerve stimulator [12].

**Fig. 3:**
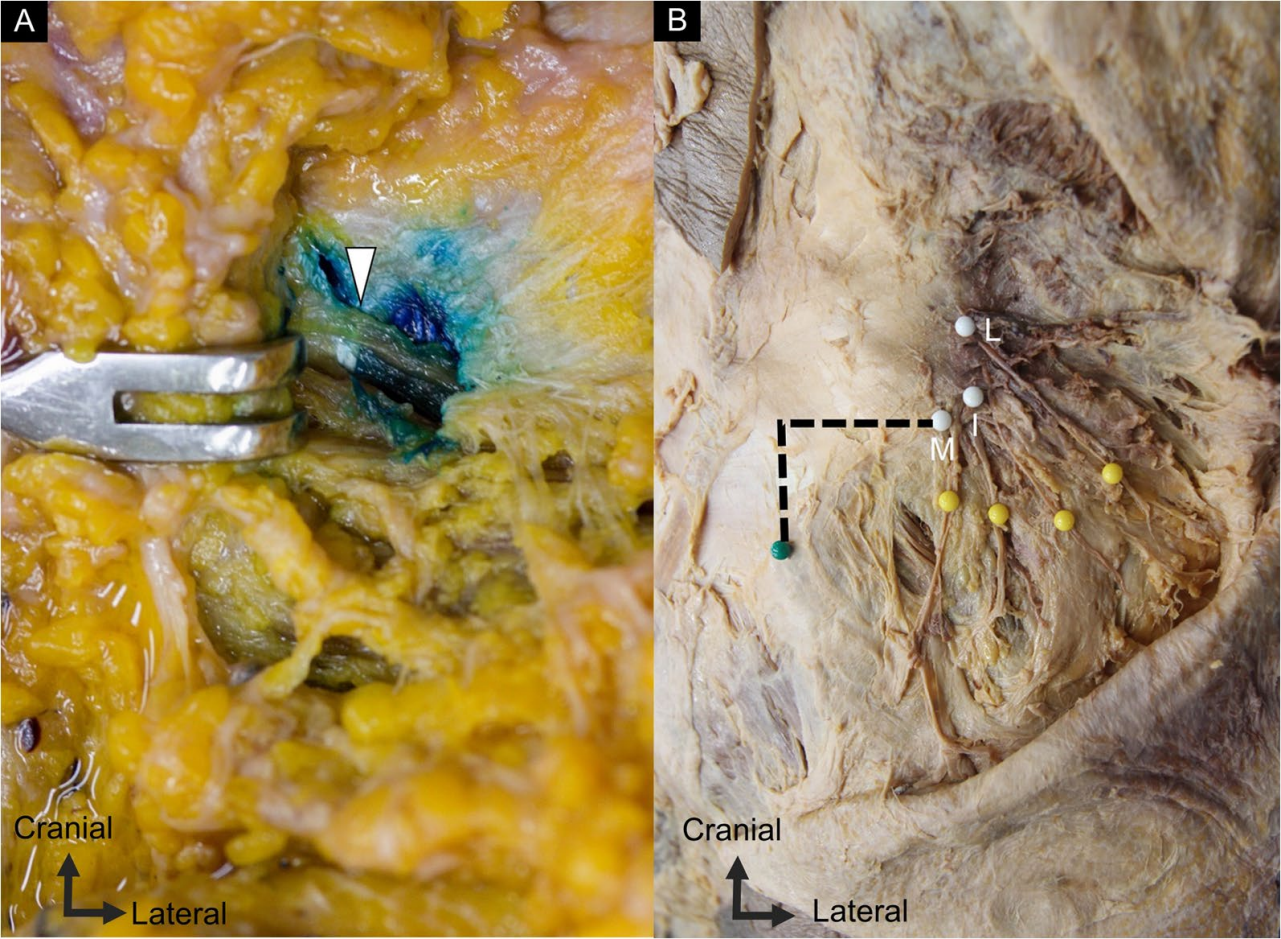
**A** The medial branch of the superior cluneal nerve was stained by methylene blue following ultrasound guided injection on the cadavers. **B** The distance between the superior cluneal nerves over the iliac crest (white dots) and the posterior superior iliac spine (green dot) was measured along the horizontal and vertical axes (dashed lines)

In patients who required ultrasound-guided nerve hydrodissection, a mixture of 1 mL 50% of dextrose, 4 mL of 1% lidocaine, and 5 mL of 0.9% normal saline was used primarily for the medial branch. Ultrasound guidance was used with an in-plane approach to target the branches of the SCN over the iliac crest. Additionally, the needle was gradually inserted using the medial-to-lateral approach to completely dissect the space between the thoracolumbar fascia and underlying bony cortex (Fig. 2F). The aforementioned injection technique was confirmed on a “Fix for Life” embalmed cadaver by successfully staining the SCN with methylene blue injection (Fig. 3A) [13].

### Track 1: dissection study

An academic anatomist with > 25 years of experience performed the dissections. Twenty sides of 10 formalin-fixed cadavers (six males and four females with an average height and age at death of 164.7 cm and 78.7 years, respectively) were dissected. Following a transverse skin incision at approximately the 12th thoracic vertebral level, the skin was removed, and the SCNs were identified above the iliac crest by blunt dissection. They were then traced from the lateral margin of the erector spinae muscle, where they penetrated the thoracolumbar fascia to the point where they crossed the iliac crest (Fig. 3B). The points at which the SCN branches crossed the iliac crest were marked, and their distances relative to the PSIS near the level of the S2 foramen were measured.

### Track 2: healthy volunteer study

Participants were enrolled if they were > 20 years and had no lower back or gluteal pain in the past year. Participants were excluded if they had previous trauma or injury to the lumbar or buttock areas. All the participants underwent bilateral clinical assessments and ultrasound scanning of the SCNs in a single session.

As the fascicles of the SCN were small, we utilized Image J software (National Institutes of Health, Bethesda, Maryland, USA) to measure their CSA, encompassing the epineurium, from the archived ultrasound images [14]. Horizontal and vertical distances of the SCNs on the iliac crest, in relation to the PSIS, were also evaluated. Additionally, a handheld algometer was used to quantify the pain threshold at the sites where the medial, intermediate, and lateral SCN branches crossed the iliac crest. The algometer was slowly and constantly applied to the target skin until the participant initially perceived tenderness with a visual analogue scale (VAS) score reaching 4 [15].

### Track 3: study of patients with SCN entrapment

Patients with unilateral symptoms were recruited from the physical medicine and rehabilitation clinic if they were > 20 years and had low back pain that radiates to the ipsilateral gluteal region, as well as unilateral tenderness on the iliac crest. Patients were excluded if they had poor control of their existing medical conditions (e.g., diabetes mellitus), rheumatic disorders, lower back injections within the past year, or previous lumbar/pelvic surgery. Plain radiographs of the lumbosacral region were obtained from each patient to exclude the possibility of bony lesions. In our study, spondylosis was operationalized as a Kellgren and Lawrence grade of ≥ 2 [16], significant spondylolisthesis was defined as the presence of vertebral slippage [17], and scoliosis was characterized by Cobb's angle 10° [18]. Furthermore, if patients were found to have other lumbar or sacral pathologies, such as lumbar herniated disk with nerve root compression or sacroiliitis, identified from recent computed tomography or magnetic resonance imaging, they would also be excluded from this study. In patients with visible posterior facet joint arthrosis, they would be categorized in the group of spondylosis. It is important to note that a patient could have more than one of these pathologies, as indicated in the registry. Once the required data was collected, an ultrasound-guided perineural injection was performed on the painful side.

The ultrasound-derived CSA, pain threshold of the SCNs, and clinical progress were evaluated at baseline and one and three months after injection. Outcome assessment comprised the VAS for the worst pain experienced over the last 24 h and the Oswestry Disability Index (ODI) [19], which evaluates pain intensity and its impact on personal care, lifting, walking, sitting, standing, sleeping, sex, social interaction, and traveling.

### Statistical analysis

Continuous variables are given as mean, standard deviation, and 95% confidence interval (CI); they were compared using the Mann–Whitney U test or visual inspection of their distribution on the blobbogram. If the 95% CIs of two effect size estimates did not overlap, they were considered significantly different [20]. Furthermore, the influence of age, sex, laterality, presence of entrapment, and being the painful site on the SCN CSA was investigated using generalized estimating equations [21], which are beneficial for analyzing data with repeated measurements or observations within the same individual or when the data are clustered by factors like location or time. Categorical variables are represented as percentages and were compared using the Chi-square test or Fisher’s exact (in the case of sparse data) test. Longitudinal data were analyzed using repeated-measures analysis of variance to examine changes over time. All analyses were conducted using SPSS 21.0 (IBM SPSS Statistics for Windows, Version 21.0, Armonk, NY, United States), and a *p*-value of < 0.05 was considered statistically significant.

## Results

### Location of the SCN on the iliac crest

**Fig. 4:**
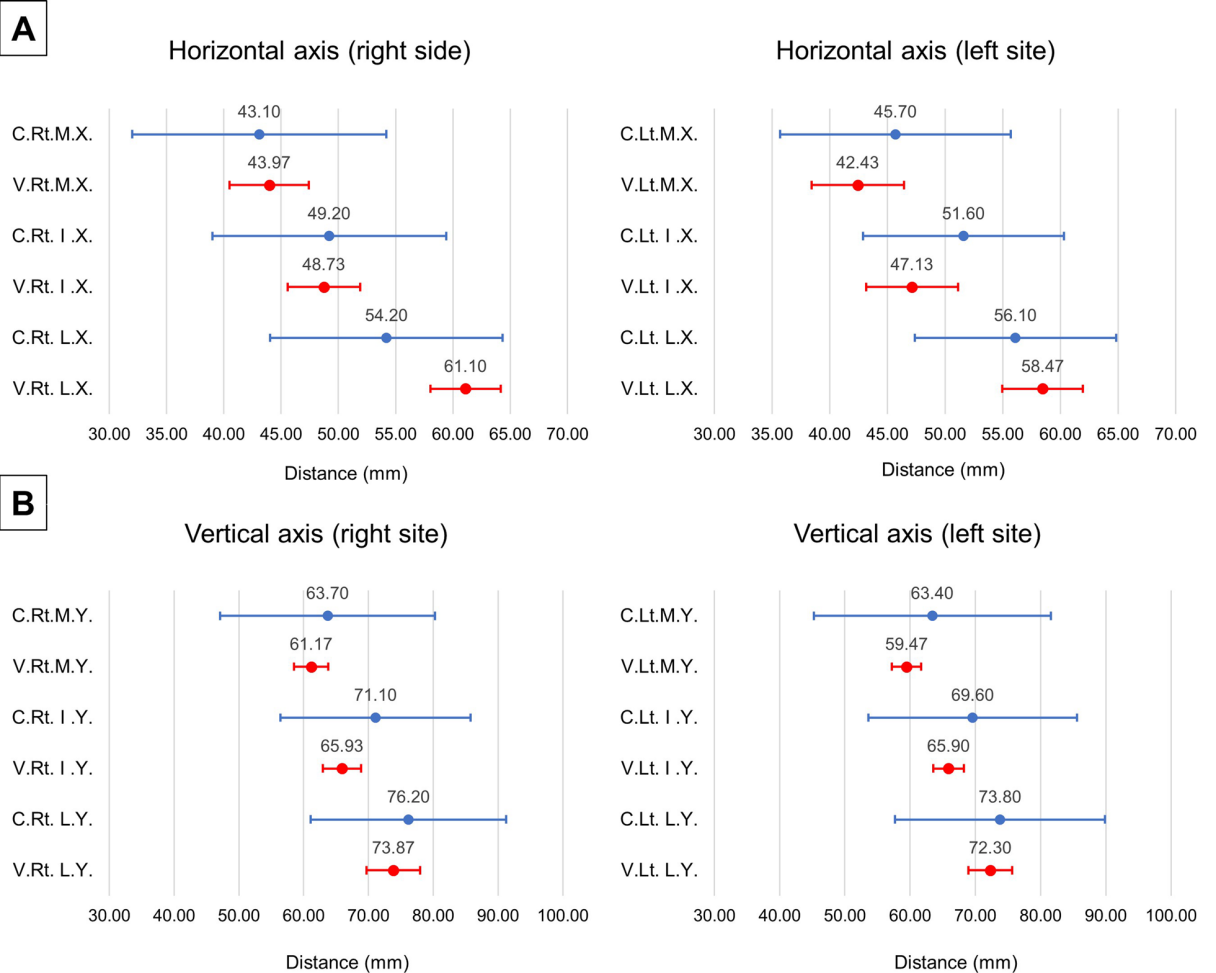
The mean value and 95% confidence interval of the distance between the superior cluneal nerves over the iliac crest and the posterior superior iliac spine on the (**A**) horizontal and (**B**) vertical axes. C., cadaver; V., asymptomatic volunteers; Rt., right; Lt., left; M., medial branch; I., intermediate branch; L., lateral branch; X., horizontal axis; Y., vertical axis

On the cadavers, the average distance between the SCN and PSIS on the horizontal axis was 43.10–45.70 mm for the medial branch, 49.20–51.60 mm for the intermediate branch, and 54.20–56.10 mm for the lateral branch. The mean distance on the vertical axis was 63.40–63.70 mm for the medial branch, 69.60–71.10 mm for the intermediate branch, and 73.80–76.20 mm for the lateral branch. The measurements were similar between healthy volunteers and cadavers (Table 1, Fig. 4).

**Table 1:** The distance between the superior cluneal nerve on the iliac crest and posterior superior iliac spine on the horizontal and vertical axes of each branch

	Cadaver (*n* = 10)			Asymptomatic volunteer (*n* = 30)		
	Right side	Left side	*p* value (R. vs. L.)	Right side	Left side	*p* value (R. vs. L.)
Medial branch						
Horizontal axis (mm)	43.10 ± 15.50 (32.02 to 54.18)	45.70 ± 13.99 (35.69 to 55.71)	0.325	43.97 ± 9.26 (40.51 to 47.42)	42.43 ± 10.74 (38.42 to 46.44)	0.217
Vertical axis (mm)	63.70 ± 23.16 (47.13 to 80.27)	63.40 ± 25.40 (45.23 to 81.57)	0.575	61.17 ± 7.04 (58.54 to 63.79)	59.47 ± 6.02 (57.22 to 61.72)	0.225
Intermediate branch						
Horizontal axis (mm)	49.20 ± 14.27 (38.99 to 59.41)	51.60 ± 12.16 (42.90 to 60.30)	0.312	48.73 ± 8.49 (45.56 to 51.90)	47.13 ± 10.65 (43.16 to 51.11)	0.493
Vertical axis (mm)	71.10 ± 20.53 (56.41 to 85.79)	69.60 ± 22.38 (53.59 to 85.61)	0.906	65.93 ± 7.91 (62.98 to 68.89)	65.90 ± 6.24 (63.57 to 68.23)	0.914
Lateral branch						
Horizontal axis (mm)	54.20 ± 14.20 (44.05 to 64.35)	56.10 ± 12.20 (47.37 to 64.83)	0.440	61.10 ± 8.21 (58.03 to 64.17)	58.47 ± 9.36 (54.97 to 61.96)	0.211
Vertical axis (mm)	76.20 ± 21.11 (61.10 to 91.30)	73.80 ± 22.51 (57.70 to 89.90)	0.953	73.87 ± 11.05 (69.71 to 77.99)	72.30 ± 8.92 (68.97 to 75.63)	0.150

The values are expressed by the mean and standard deviation (95% confidence interval of mean). R., right; L., left

### Cross-sectional area of the SCN

The average nerve CSA at baseline ranged between 4.69–5.67 mm^2^ across different branches and sides (Table 2). Generalized estimating equations analysis showed that age, sex, laterality, presence of entrapment, and site of pain did not have a significant impact on nerve size (Table 3). Furthermore, there was no significant difference (*p* = 0.982) in entrapped SCN CSA at the iliac crest level between individuals responding to injections (4.94 ± 1.20 mm^2^; 95% CI, 4.45 to 5.44) and those not responding to injections (5.13 ± 1.83 mm^2^; 95% CI, 3.44 to 6.83).

**Table 2:** Cross-sectional area of the superior cluneal nerve at different branches and sides

	Asymptomatic volunteers (*n* = 30)			Patients (*n* = 36)		
	Right side	Left side	*p* value (R. vs. L.)	Painful side	Non-painful side	*p* value (P. vs. N.)
Cross-sectional area (mm^2^)						
Medial branch						
Baseline	5.27 ± 1.99 (4.51 to 6.02)	4.88 ± 1.95 (4.15 to 5.61)	0.094	5.04 ± 1.34 (4.57 to 5.50)	4.93 ± 1.32 (4.43 to 5.42)	0.125
1st post-injection month	–	–	–	5.32 ± 1.57 (4.78 to 5.86)	5.13 ± 1.40 (4.65 to 5.61)	0.555
3rd post-injection month	–	–	–	5.44 ± 1.86 (4.80 to 6.08)	5.09 ± 1.32 (4.63 to 5.54)	0.287
Intermediate branch						
Baseline	5.08 ± 1.96 (4.34 to 5.83)	4.95 ± 2.03 (4.20 to 5.71)	0.284	5.28 ± 1.72 (4.68 to 5.88)	5.29 ± 1.52 (4.73 to 5.86)	0.513
1st post-injection month	–	–	–	5.37 ± 1.66 (4.80 to 5.94)	5.25 ± 1.39 (4.77 to 5.73)	0.471
3rd post-injection month	–	–	–	5.35 ± 1.25 (4.91 to 5.78)	5.35 ± 1.51 (4.83 to 5.87)	0.719
Lateral branch						
Baseline	4.90 ± 2.14 (4.08 to 5.71)	4.69 ± 2.02 (3.94 to 5.45)	0.336	5.56 ± 1.43 (5.06 to 6.06)	5.67 ± 1.48 (5.11 to 6.22)	0.272
1st post-injection month	–	–	–	5.59 ± 1.46 (5.09 to 6.09)	5.78 ± 1.82 (5.15 to 6.41)	0.376
3rd post-injection month	–	–	–	5.52 ± 1.35 (5.06 to 5.98)	5.55 ± 1.52 (5.03 to 6.08)	0.838

The values are expressed by the mean and standard deviation (95% confidence interval of mean). R., right; L., left; P., painful; N., non-painful

**Table 3:** Association of the cross-sectional area of the SCN with clinical characteristics

	Cross-sectional area		
	Medial branch	Intermediate branch	Lateral branch
Female	−0.557 (−1.298 to 0.185)	−0.409 (−1.201 to 0.384)	−0.641 (−1.444 to 0.162)
	*p* = 0.141	*p* = 0.312	*p* = 0.118
Age	0.021 (0.021 to 0.047)	0.021 (0.021 to 0.048)	0.020 (0.020 to 0.047)
	*p* = 0.096	*p* = 0.135	*p* = 0.152
Right site	0.006 (0.006 to 0.425)	−0.089 (−0.089 to 0.405)	−0.009 (−0.009 to 0.405)
	*p* = 0.978	*p* = 0.724	*p* = 0.967
Patients with pain^a^	−0.209 (−0.209 to 0.617)	0.161 (0.161 to 1.055)	0.816 (0.816 to 1.700)
	*p* = 0.620	*p* = 0.725	*p* = 0.071
Side of Pain	0.119 (0.119 to 0.709)	0.020 (0.020 to 0.713)	−0.094 (−0.094 to 0.489)
	*p* = 0.691	*p* = 0.956	*p* = 0.751

The values are expressed by β coefficients and their 95% confidence intervals

^a^Indicates that the control group is taken as the reference

### Pain pressure threshold on the iliac crest

There was no significant difference in the pressure threshold for pain between the right and left sides of the three branches of the SCN in asymptomatic volunteers. However, in patients with entrapment, a significantly lower threshold was observed on the painful side than on the asymptomatic side for all three branches (Additional file 1: Table S1).

### Outcome of ultrasound-guided injection

A comparison of the demographics of asymptomatic volunteers and patients with SCN entrapment is presented in Additional file 1: Table S2. Among the 36 patients, a significant reduction in the VAS and ODI scores, along with an increase in the pressure threshold of pain on the injected side, was observed in the first and third months after the injection (Additional file 1: Table S3). However, there was no change in nerve CSA. The initial treatment success (i.e., VAS change > 1 mm at the first post-injection month) [22] was observed in 77.7% (*n* = 28) of the patients. Patients with treatment failure had lower baseline VAS scores than those with initial treatment success (Additional file 1: Table S4).

**Fig. 5:**
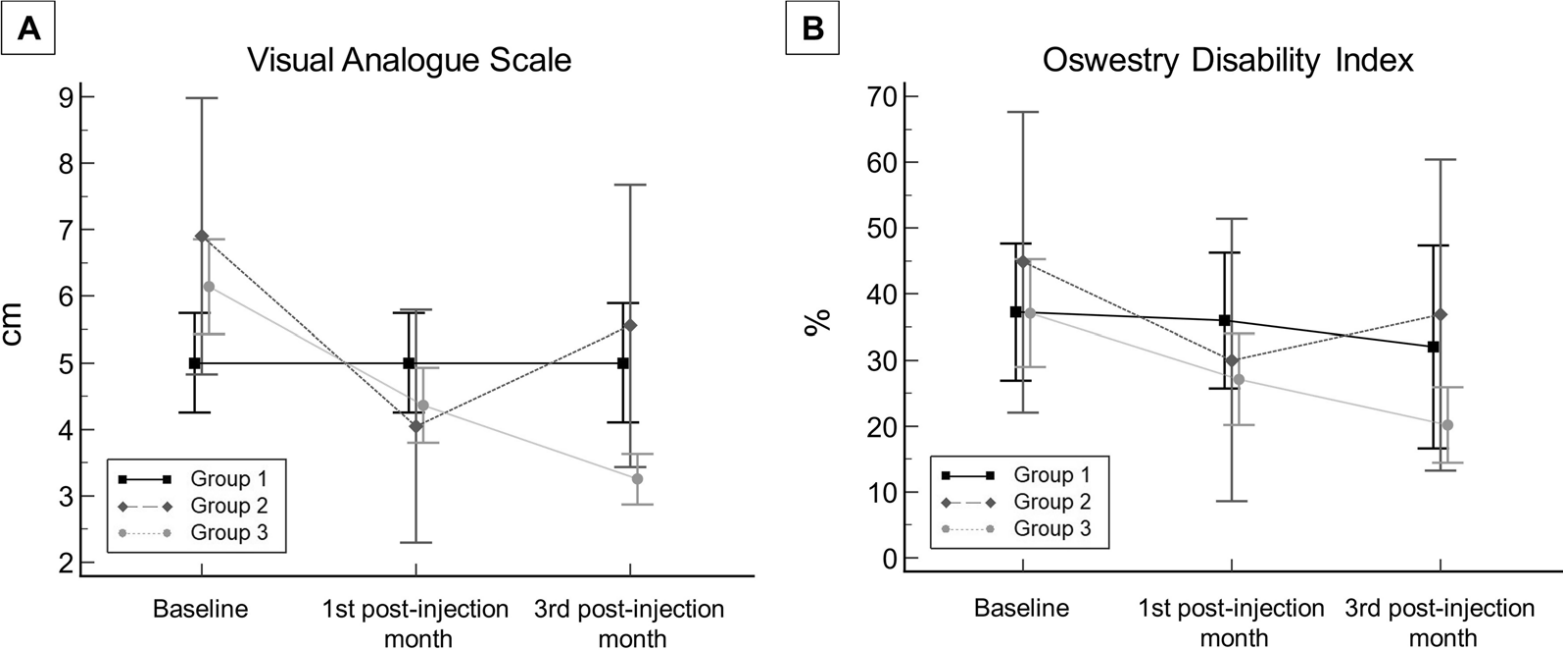
**A** Visual analogue scale of pain and (**B**) Oswestry Disability Index at baseline, post-injection 1st and 3rd months for each group. Group 1, who did not respond to the initial treatment; Group 2, who initially responded to treatment but experienced symptom recurrence; and Group 3, who responded well to treatment and did not experience symptom recurrence

Symptom recurrence (i.e., third-month VAS ≥ baseline VAS) was observed in 25.0% (*n* = 7) of the patients with initial treatment success (Fig. 5). The group with recurrent pain had a higher prevalence of scoliosis than the group without recurrent pain (57.14% vs. 0.00%, *p* = 0.002; Table 4). None of the patients experienced any post-intervention adverse events.

**Table 4:** Comparison between baseline characteristics of patients with and without symptom recurrence

	Symptom recurrence (−) (*n* = 21)	Symptom recurrence (+) (*n* = 7)	*p* value
Female (%)	14 (66.67%)	4 (57.14%)	0.491
Age (year)	60.19 ± 11.74 (54.85 to 65.54)	62.29 ± 13.20 (50.08 to 74.49)	0.695
Height (cm)	160.07 ± 8.81 (155.83 to 164.32)	157.90 ± 6.05 (151.55 to 164.25)	0.642
Weight (kg)	61.69 ± 11.16 (56.31 to 67.07)	57.93 ± 10.64 (46.77 to 69.10)	0.475
Smoke (%)	0 (0.00%)	0 (0.00%)	1.000
Alcohol (%)	1 (4.76%)	0 (0.00%)	0.750
Diabetes mellitus (%)	0 (0.00%)	0 (0.00%)	1.000
Spondylosis (%)	5 (23.81%)	1 (14.29%)	0.522
Spondylolisthesis (%)	2 (9.52%)	0 (0.00%)	0.556
Scoliosis (%)	0 (0.00%)	4 (57.14%)	0.002*
Visual Analogue Scale at Baseline (cm)	6.14 ± 1.48 (5.43 to 6.85)	6.90 ± 2.24 (4.83 to 8.98)	0.364
Oswestry Disability Index at Baseline (%)	37.10 ± 16.90 (28.95 to 45.25)	44.98 ± 24.51 (22.31 to 67.66)	0.360

*Indicates *p* < 0.05. The values of continuous variables are expressed by the mean and standard deviation (95% confidence interval of mean). The values of categorical variables are expressed by the number (percentage)

## Discussion

This study yielded several important results. First, the SCN locations crossing the iliac crest did not differ between the cadavers and asymptomatic volunteers, as confirmed by ultrasound imaging and electrical stimulation. Second, the CSA of the SCN did not vary across branches or in the presence of pain. Third, the average pain threshold decreased in all three SCN branches on the painful side. Fourth, approximately 80% of the patients responded successfully to ultrasound-guided 5% dextrose hydrodissection, whereas the rate of symptom recurrence was up to 25% in those with initial injection success.

The location of the SCN on the iliac crest has been previously delineated in cadaveric studies. In 2015, Loubser et al. [18] investigated 27 cadavers and reported that the average distance between the SCN piercing the thoracolumbar fascia and PSIS was 69.6 mm. In another study, Iwanaga et al. [19] dissected 10 freshly frozen cadavers and found that the mean distance from the bony groove on the iliac crest containing the medial SCN branch to the PSIS was 45.2 mm. In our cadaveric study, we specifically measured the distance between the above-crest SCN and PSIS along the horizontal and vertical axes to differentiate the reciprocal positions of different SCN branches on the same side. Using our measurement protocol, we found that the observations in cadavers were similar to the ultrasound findings in asymptomatic volunteers regarding the locations of all three SCN branches. This finding partially implies the validity of our sonographic approach. Furthermore, according to a recent systematic literature review [5], there is a lack of ultrasound studies investigating the precise location of the SCN on the iliac crest. In our investigation, we relied on a report describing the median branch of the SCN [23], which was observed to be embedded within the osteofibrous tunnel on the iliac crest.

Our study also revealed that ultrasonography-determined CSA has little value in the diagnosis of SCN entrapment. The use of CSA for diagnosing nerve entrapment is most evident in carpal and cubital tunnel syndromes, where the nerves usually become enlarged proximal to the compression site because of obstruction of the axoplasmic flow [24, 25]. However, unlike the median and ulnar nerves, which have considerable diameters owing to multiple intra-neural bundles, the SCN (being a cutaneous nerve) is small and has only a single nerve bundle [26]. The epineurium of the SCN contains a thick hyperechoic layer of adipose tissue, rendering the hypoechoic nerve bundles insignificant in terms of nerve CSA. Therefore, even if the nerve bundles become swollen because of entrapment, it is still challenging to differentiate them using ultrasound imaging. Our findings were consistent with those of an early case series comprising nine patients with SCN entrapment [6], which revealed significant nerve enlargement only in one patient after posterior lumbar interbody fusion.

Because tenderness over the iliac crest is considered mandatory for diagnosis, a decreased pain pressure threshold over the affected site is expected in SCN entrapment. It is important to note that increased pain sensitivity over the painful site was observed in all three branches of the nerve and not just in the medial branch. Tension of the thoracolumbar fascia is a common cause of SCN entrapment [27, 28]. The medial branch, which typically passes through a narrow fibro-osseous tunnel on the iliac crest, is most susceptible to compression; intermittent and lateral branches can also be affected, particularly in the segment that pierces the thoracolumbar fascia. Therefore, in patients with SCN entrapment, treatment with injections should include hydrodissection of the thoracolumbar fascia to maximize therapeutic effectiveness.

We observed an approximately 80% response rate to ultrasound-guided nerve hydrodissection, but several factors may have contributed to the remaining failed cases. For instance, the SCN could have been entrapped at multiple levels, such as inside the erector spinae muscle or in the subcutaneous layer, whereas our approach only targeted segments crossing the iliac crest. Additionally, ultrasound-guided intervention may not correct faulty biomechanics, such as excessive lumbar lordosis, which has been found to accentuate SCN entrapment. Finally, the SCN is in proximity to many common pain generators in the lumbosacral region, such as the lumbar facets, sacroiliac joints, and gluteus muscle insertions. Pathologies in these locations can generate symptoms similar to or concurrent with SCN entrapment.

Recurrent pain was observed in 25% of the patients who initially responded to the injection, and scoliosis was associated with recurrent pain after the initial treatment success. Scoliosis can cause asymmetry in the tension of the thoracolumbar fascia, which can alter the biomechanics of the spine and the surrounding muscles. An observational study also suggested that continued tightening of the fascial plane due to asymmetrical tension distribution could further increase scoliotic curvature [29]. Our findings indicate that although initial hydrodissection effectively relieves the compressed SCN, nerves may become repeatedly entrapped due to unbalanced posture factors. However, it is important to note that the exact causal relationship between recurrent pain and scoliosis requires further validation through large-scale studies.

For nerve hydrodissection, we used an injectate consisting of 5% dextrose, 1% lidocaine, and normal saline. Various regimens, including corticosteroids, platelet-rich plasma, and 5% dextrose, are commonly used for nerve hydrodissection. In case of carpal tunnel syndrome, a network meta-analysis has demonstrated that 5% dextrose is the most effective injectate for symptom relief [30]. Additionally, choosing 5% dextrose enables repeat injections at short intervals, which is otherwise not possible with corticosteroids because of their adverse effects on adjacent soft tissues. Furthermore, the long-term effect of 5% dextrose injection has been implied by a previous randomized controlled trial [31], showing 5% dextrose to be more effective than corticosteroid injection in patients with mild-to-moderate carpal tunnel syndrome at 4 to 6 months post-injection. Our study also implies that repeat injections, such as hydrodissection along the nerve’s long axis (to cover the subcutaneous or intramuscular segments), may be needed in cases of initial treatment failure or when using the same injection approach for symptom recurrence in patients with primary treatment success.

This study has several limitations. First, the injection techniques were validated at only two sites on a cadaver. This was due to the fact that if the methylene blue staining was used on the other 10 cadavers, the exact locations of the SCN on the iliac crest might have not been accurately located. Second, we did not administer placebo injections over the subcutaneous layer or other sham treatments for comparison with the patient group. Our clinical objective was to investigate the responsiveness and predictors of ultrasound-guided 5% dextrose hydrodissection for SCN entrapment; therefore, future studies are needed to explore the comparative effectiveness of our approach with other treatment methods. Third, structured rehabilitation was not incorporated after the injection. Considering that scoliosis is associated with symptom recurrence, it may be necessary to include physical therapy and exercise interventions in standard post-injection care to reduce thoracolumbar fascial tension and improve pelvic symmetry. Fourth, our study only followed patients for a period of three months, leaving the long-term effects of ultrasound-guided intervention unknown. Additionally, our strict inclusion criteria excluded patients with nonspecific low back pain who did not exhibit tenderness over the iliac crest. Future studies are necessary to explore the duration of the effect of ultrasound-guided SCN hydrodissection and determine whether the benefits of this intervention can extend to a broader patient population beyond those with SCN entrapment alone. Fifth, we did not routinely arrange all patients with initial injection failure or recurrent pain for EOS imaging [32], magnetic resonance imaging, or computed tomography. It is possible that unsuccessful treatment or symptom recurrence could be attributed to mild/moderate facet arthrosis or sacroiliac joint pathology, which would require the aforementioned advanced imaging systems for investigation.

## Conclusion

Ultrasound imaging enables the localization of SCN branches on the iliac crest, and the findings are similar to those of cadaveric dissections. Enlargement of the nerve CSA is not useful for diagnosing SCN entrapment, and approximately 80% of cases respond positively to ultrasound-guided dextrose hydrodissection. Scoliosis is associated with symptom recurrence in patients with successful initial injections, and whether structured rehabilitation can reduce recurrence post-injection should be considered as one perspective in future research.

## Supplementary Information

**Additional file 1.** Supplemental tables.

## Data Availability

The datasets used and/or analyzed during the current study are available from the corresponding author on reasonable request.

## Abbreviations

CI: Confidence interval
CSA: Cross-sectional area
ODI: Oswestry Disability Index
PSIS: Posterior superior iliac spine
SCN: Superior cluneal nerve
VAS: Visual analog scale
