# Supplementary material for: Enhancing diagnosis and treatment of superior cluneal nerve entrapment: cadaveric, clinical, and ultrasonographic insights
Source: Insights Imaging. 2023 Jul 3;14:116. doi: 10.1186/s13244-023-01463-0 (PMC10317927; doi:10.1186/s13244-023-01463-0)
Supplement: Supplementary file 1 — Additional file 1. Supplemental tables. [file 13244_2023_1463_MOESM1_ESM.pdf]

ELECTRONIC SUPPLEMENTARY MATERIAL

Table S1. Baseline pain pressure threshold on the iliac crest of each branch of the superior cluneal nerve

	Asymptomatic volunteers (n =30)			Patients (n =36)		
	Right side	Left side	*p* value (R. vs. L.)	Painful side	Non-painful side	*p* value (P. vs N.)
Pain pressure threshold (kg)						
Medial branch	4.53 ± 0.41 (4.38 to 4.68)	4.51 ± 0.52 (4.32 to 4.71)	0.766	4.30 ± 0.67 (4.06 to 4.54)	5.08 ± 0.48 (4.91 to 5.26)	**<0.001***
Intermediate branch	4.66 ± 0.47 (4.49 to 4.84)	4.54 ± 0.52 (4.34 to 4.73)	0.122	4.50 ± 0.65 (4.26 to 4.73)	4.99 ± 0.60 (4.77 to 5.20)	**<0.001***
Lateral branch	4.70 ± 0.42 (4.54 to 4.85)	4.57 ± 0.67 (4.32 to 4.82)	0.154	4.47 ± 0.63 (4.25 to 4.70)	4.82 ± 0.72 (4.56 to 5.08)	**0.003***

* Indicates $p < 0.05$. The values are expressed by the mean and standard deviation (95% confidence interval of mean). R., right; L., left; P., painful side; N., non-painful side.

Table S2. Basic characteristics between the asymptomatic volunteers and patients with superior cluneal nerve entrapment

	Asymptomatic Volunteers (n =30)	Patients (n =36)	*p* value
Female (%)	14 (46.67%)	23 (63.89%)	0.215
Age (year)	49.70 ± 15.81 (43.80 to 55.60)	60.33 ± 13.36 (55.81 to 64.85)	**0.004***
Height (cm)	164.64 ± 8.54 (161.19 to 168.09)	160.51 ± 8.61 (157.35 to 163.67)	0.076
Weight (kg)	65.91 ± 12.04 (61.05 to 70.77)	62.77 ± 11.58 (58.60 to 66.95)	0.318
Smoke (%)	3 (10.00%)	0 (0.00%)	0.089
Alcohol (%)	1 (3.33%)	1 (2.78%)	1.000
Diabetes mellitus (%)	1 (3.33%)	1 (2.78%)	1.000

Spondylosis (%)	0<br>(0.00%)	9<br>(25.00%)	**0.003***
Spondylolisthesis (%)	0<br>(0.00%)	3<br>(8.33%)	0.245
Scoliosis (%)	0<br>(0.00%)	5<br>(13.89%)	0.058
Visual Analogue Scale at Baseline (cm)	0.00 ± 0.00<br>(0.00 to 0.00)	6.03 ± 1.64<br>(5.46 to 6.60)	**<0.001***
Oswestry Disability Index at Baseline (%)	10.29 ± 15.98<br>(4.32 to 16.26)	38.77 ± 17.56<br>(32.64 to 44.90)	**<0.001***

* Indicates $p < 0.05$. The values of continuous variables are expressed by the mean and standard deviation (95% confidence interval of mean). The values of categorical variables are expressed by the number (percentage).

Table S3. Cross-sectional area of the superior cluneal nerve, pain pressure threshold, and clinical assessment values

	Time Points of Measurement			
	baseline	1st post-injection month	3rd post-injection month	*p* value (repeated measured AONVA)
Cross-sectional area at the painful side (mm^2)				
Medial branch	5.04 ± 1.34 (4.57 to 5.50)	5.32 ± 1.57 (4.78 to 5.86)	5.44 ± 1.86 (4.80 to 6.08)	0.176
Intermediate branch	5.28 ± 1.72 (4.68 to 5.88)	5.37 ± 1.66 (4.80 to 5.94)	5.35 ± 1.25 (4.91 to 5.78)	0.658
Lateral branch	5.56 ± 1.43 (5.06 to 6.06)	5.59 ± 1.46 (5.09 to 6.09)	5.52 ± 1.35 (5.06 to 5.98)	0.859
Pain pressure threshold at the painful side (kg)				
Medial branch	4.30 ± 0.67[ab] (4.06 to 4.54)	5.40 ± 0.56[a] (5.21 to 5.60)	5.40 ± 0.53[b] (5.21 to 5.58)	**<0.001***
Intermediate branch	4.50 ± 0.65[ab] (4.26 to 4.73)	5.16 ± 0.98[a] (4.82 to 5.49)	5.43 ± 0.45[b] (5.27 to 5.59)	**<0.001***
Lateral branch	4.47 ± 0.63[ab] (4.25 to 4.70)	5.17 ± 0.67[a] (4.94 to 5.40)	5.27 ± 0.42[b] (5.12 to 5.42)	**<0.001***
Clinical assessment				
Visual Analogue Scale	6.03 ± 1.64[ab] (5.46 to 6.60)	4.44 ± 1.32[a] (4.00 to 4.89)	4.02 ± 1.49[b] (3.50 to 4.54)	**<0.001***
Oswestry Disability Index (%)	38.77 ± 17 56[ab] (32.64 to 44.90)	29.69 ± 16.35[a] (24.16 to 35.23)	25.56 ± 16.53[b] (19.80 to 31.33)	**0.001***

* Indicates $p < 0.05$. The values were expressed by the mean and standard deviation (95% confidence interval of mean). [a] significant difference between baseline and the 1st post-injection month in the same group. [b] significant difference between baseline and the 3rd post-injection month in the same group. [c] significant difference between the 1st post-injection month and 3rd post-injection month in the same group. ANOVA, analysis of variance.

Table S4. Baseline characteristics of patients with and without initial treatment success

	Initial treatment success (+) (n =28)	Initial treatment success (-) (n =8)	*p* value
Female (%)	18 (64.29%)	5 (62.50%)	0.618
Age (year)	60.71 ± 11.90 (56.10 to 65.33)	59.00 ± 18.53 (43.51 to 74.49)	0.754
Height (cm)	159.55 ± 8.17 (156.18 to 162.92)	164.50 ± 10.01 (153.99 to 175.01)	0.364
Weight (kg)	60.79 ± 10.94 (56.28 to 65.30)	69.86 ± 11.82 (58.92 to 80.79)	0.066
Smoke (%)	0 (0.00%)	0 (0.00%)	1.000
Alcohol (%)	1 (3.57%)	0 (0.00%)	0.778
Diabetes mellitus (%)	0 (0.00%)	1 (12.50%)	0.222
Spondylosis (%)	6 (21.43%)	3 (37.50%)	0.310
Spondylolisthesis (%)	2 (7.14%)	1 (12.50%)	0.541
Scoliosis (%)	4 (14.29%)	1 (12.50%)	0.695
Baseline Visual Analogue Scale at Baseline (cm)	6.35 ± 1.70 (5.66 to 7.03)	5.00 ± 0.89 (4.26 to 5.74)	**0.047***

Oswestry Disability Index at Baseline (%)	39.22 ± 19.04 (31.53 to 46.91)	37.31 ± 12.45 (26.89 to 47.72)	0.792

* Indicates *p* <0.05. The values of continuous variables are expressed by the mean and standard deviation (95% confidence interval of mean). The values of categorical variables are expressed by the number (percentage).
